# *Notes from the Field:* Ongoing Cluster of Highly Related Disseminated Gonococcal Infections — Southwest Michigan, 2019

**DOI:** 10.15585/mmwr.mm6912a5

**Published:** 2020-03-27

**Authors:** William D. Nettleton, James B. Kent, Kathryn Macomber, Mary-Grace Brandt, Kelly Jones, Alison D. Ridpath, Brian H. Raphael, Eden V. Wells

**Affiliations:** ^1^Kalamazoo County Health and Community Services Department, Kalamazoo, Michigan; ^2^Department of Family and Community Medicine, Western Michigan University Homer Stryker M.D. School of Medicine, Kalamazoo, Michigan; ^3^Michigan Department of Health and Human Services; ^4^Division of STD Prevention, National Center for HIV/AIDS, Viral Hepatitis, STD, and TB Prevention, CDC.

Disseminated gonococcal infection is a rare, systemic complication of untreated gonorrhea that occurs after sexual transmission and through hematogenous spread of *Neisseria gonorrhoeae* to distant body sites (*1*). Disseminated gonococcal infection usually manifests as arthritis, dermatitis, and tenosynovitis. In rare cases, endocarditis, meningitis, myositis, and osteomyelitis can occur. On August 12, 2019, the Kalamazoo County Health and Community Services Department (KCHCSD), Michigan, was notified of three persons hospitalized with disseminated gonococcal infection. Given the rarity of disseminated gonococcal infection, severe case presentations, and ongoing case clustering, KCHCSD and the Michigan Department of Health and Human Services (MDHHS) initiated a joint investigation. Actions included health alerts and public notifications, medical record reviews, patient interviews, antimicrobial resistance testing, and whole genome sequencing (WGS) of *N. gonorrhoeae* isolates by MDHHS and CDC laboratories. A review of approximately 27,000 gonorrhea cases from the preceding 18 months revealed no other location or time clustering of disseminated gonococcal infection in Michigan. To better characterize the cluster, case definitions were developed.

A confirmed case was defined as isolation of *N. gonorrhoeae* from any sterile site, including blood, synovial fluid, or cerebrospinal fluid. A probable case was defined as a positive nucleic acid amplification test from nonsterile sites (e.g., urethra, vagina, cervix, rectum, or pharynx) in the presence of signs or symptoms (e.g., tenosynovitis or polyarthralgias). Thirteen confirmed and three probable cases were reported during August 12–December 18, 2019. Fourteen of these 16 patients resided in Kalamazoo County and two in bordering southwestern Michigan counties.

Nine of the 16 patients were male, and patient ages ranged from 16 to 52 years (Table). Patients initially had one or more of the following manifestations: septic arthritis (13 patients), myositis (four), tenosynovitis (three), osteomyelitis (two), and mitral valve endocarditis (one). Only the patient with endocarditis had *N. gonorrhoeae* isolated from blood. Fifteen of the 16 patients were hospitalized, and 13 required invasive surgical intervention. Eleven had laboratory confirmation of *N. gonorrhoeae* from nondisseminated sites on initial evaluation, including eight urogenital, five pharyngeal, and one rectal. Fourteen patients received intravenous or intramuscular ceftriaxone treatment (seven patients received 4–6 weeks’ therapy). No underlying immunosuppressive disorders (e.g., human immunodeficiency virus infection or complement deficiency) or use of immunocompromising medications were identified. Four patients were homeless. Thirteen reported or tested positive for drug use (methamphetamine [10], marijuana [six], and opioids [three]), including three who reported injection drug use. Although each patient named from zero to five sex or needle-sharing partners for a total of 27 partners, interviews did not reveal direct sex or needle contact between patients within the cluster. Of 11 isolates recovered from sterile sites, all were sensitive to azithromycin, ceftriaxone, and cefixime. Despite an inability to identify social connections, WGS revealed highly related isolates, differing by 10–48 single nucleotide polymorphisms.

**TABLE:** Characteristics of patients with disseminated gonococcal infection — southwest Michigan, 2019

Characteristic	No. (%)
**Total**	**16 (100)**
Median age (yrs) (range)	39 (16–52)
**Sex**	
Male	9 (56)
Female	7 (44)
**Case status**	
Confirmed	13 (81)
Probable	3 (19)
**Residence**	
Kalamazoo County	14 (88)
Other southwest Michigan counties	2 (13)
Homeless	4 (25)
**Initial clinical manifestations**	
Septic arthritis	13 (81)
Myositis	4 (25)
Tenosynovitis	3 (19)
Osteomyelitis	2 (13)
Mitral valve endocarditis*	1 (6)
**Concurrent gonococcal infections**	11 (69)
Urogenital	8 (50)
Pharyngeal	5 (31)
Rectal	1 (6)
Hospitalized	15 (94)
Parenteral ceftriaxone treatment^†^	14 (88)
**Reported drug use or positive drug test**	13 (81)
Methamphetamine	10 (63)
Marijuana	6 (38)
Opioids	3(19)
Injection drug use	3 (19)

* This patient had *Neisseria gonorrhoeae* isolated from blood.

^†^ Seven patients received treatment for 4–6 weeks.

The clinical severity, high relatedness of isolates, and reported methamphetamine use among patients raise unique questions about host and pathogen factors that warrant further investigation. Prompt diagnosis and treatment of disseminated gonococcal infection might prevent severe disease and complications. Outreach continues to ensure case finding, clinician awareness, partner elicitation, and broad distribution of prevention messages. Enhanced surveillance, thorough investigation, and continued partnerships remain crucial for rapid identification, improved understanding, and mitigation of disseminated gonococcal infection cases and clusters identified in Michigan.
